# A Novel and Effective Method for Congestive Heart Failure Detection and Quantification Using Dynamic Heart Rate Variability Measurement

**DOI:** 10.1371/journal.pone.0165304

**Published:** 2016-11-11

**Authors:** Wenhui Chen, Lianrong Zheng, Kunyang Li, Qian Wang, Guanzheng Liu, Qing Jiang

**Affiliations:** 1 School of Engineering, Sun Yat-sen University, Guangzhou, Guangdong, China; 2 Science and Technology Planning Project of Guangdong Province, Guangzhou, Guangdong, China; 3 Guangdong Provincial Engineering and Technology Centre of Advanced and Portable Medical Device, Guangzhou, Guangdong, China; The Pennsylvania State University, UNITED STATES

## Abstract

Risk assessment of congestive heart failure (CHF) is essential for detection, especially helping patients make informed decisions about medications, devices, transplantation, and end-of-life care. The majority of studies have focused on disease detection between CHF patients and normal subjects using short-/long-term heart rate variability (HRV) measures but not much on quantification. We downloaded 116 nominal 24-hour RR interval records from the MIT/BIH database, including 72 normal people and 44 CHF patients. These records were analyzed under a 4-level risk assessment model: no risk (normal people, N), mild risk (patients with New York Heart Association (NYHA) class I-II, P1), moderate risk (patients with NYHA III, P2), and severe risk (patients with NYHA III-IV, P3). A novel multistage classification approach is proposed for risk assessment and rating CHF using the non-equilibrium decision-tree–based support vector machine classifier. We propose dynamic indices of HRV to capture the dynamics of 5-minute short term HRV measurements for quantifying autonomic activity changes of CHF. We extracted 54 classical measures and 126 dynamic indices and selected from these using backward elimination to detect and quantify CHF patients. Experimental results show that the multistage risk assessment model can realize CHF detection and quantification analysis with total accuracy of 96.61%. The multistage model provides a powerful predictor between predicted and actual ratings, and it could serve as a clinically meaningful outcome providing an early assessment and a prognostic marker for CHF patients.

## Introduction

Congestive heart failure (CHF) is a common chronic cardiovascular syndrome along with autonomic nervous system (ANS) abnormality of the heart [1]. Patients experience no obvious symptoms during its early stages. Once diagnosed, physicians still cannot provide convenient suitable medical care based on prognosis according to the patient’s physical condition. Furthermore, poor prognosis results in 30–40% of diagnosed patients dying in a year [2]. Thus, risk assessment of CHF is essential for saving lives and money. The severity of CHF has a well-known measurement, namely, the symptomatic classification scale of the New York Heart Association (NYHA) [3], which has proved to be a very useful factor for risk assessment of CHF patients [4].

According to the NYHA classification, the severity scale of heart failure depends on the severity of symptoms [5], which are partly modulated by the autonomic nervous system. Heart rate variability (HRV) analysis has been confirmed as a reliable and noninvasive tool in the prognosis and risk assessment of CHF, and it is widely used to assess the influence of the ANS on the heart [6]. HRV measurements (time/frequency domain and non-linear) of 5-minute/24-hour (5-min/24-h) data have already been studied in statistic difference levels between normal people and CHF patients [7]–[9]. Measurements of adverse changes in the autonomic function of CHF manifest in altered HRV analysis [10]. In this paper, we redefined short-/long-term (i.e., 5-min/24-h) HRV measurements as static indices (SI) to assess the autonomic function of the recording.

As far back as 1996, the Task Force of the European Society of Cardiology and the North American Society of Pacing and Electrophysiology published standards on statistical analysis of short-/long-term HRV measurements [6]. In 2003, Asyali et al. applied Bayesian classifiers to classical time/frequency HRV parameters of long-term measurements for CHF discrimination with an accuracy of 93.24% [11]. In 2007, Isler et al. utilized wavelet entropy and classical HRV parameters with k–nearest-neighbor (KNN) classifiers for CHF diagnosis and achieved an accuracy of 96.39% [12]. In 2011, Pecchia et al. applied two additional non-standard measures—ΔAVNN (average of RR intervals) and ΔLF/HF (average of LF/HF)—in CHF detection with an accuracy of 96.4% [13]. In 2012, Yu et al. applied a support vector machine (SVM) classifier and genetic algorithm (GA) into CHF recognition based on bi-spectral HRV analysis and achieved an accuracy of 98.79% [14]. These studies mainly focused on the overall level condition of autonomic function by static indices of HRV measurements for disease detection; however, relatively little attention have been paid to assessing the autonomic activity change among CHF patients. Among the results, many of these reports could distinguish CHF patients from normal people with accuracies of more than 95%. This is consistent with the fact that the redefined SI can discern the autonomic dysfunction of CHF patients from normal function [10].

By 2013, Melillo et al. first tried to assess the severity of CHF disease by using long-term HRV measurements. The classification and regression tree (CART) classifier was used to separate lower-risk patients from higher-risk patients with a relatively low accuracy (i.e., 85.4%) [15]. Two reasons may explain this result. First, the performance of the classifier needed to be improved. Second, the static HRV measurement might not fully quantify trend changes in the autonomic activity of CHF patients during different daily activity [10]. Thus, we proposed a new measurement of HRV—dynamic indices (DI)—for the stratifying estimate. DI reflects the dynamic of 5-min segments’ HRV measurement in 24 hours, described in HRV measurement Part. The functional class of CHF patients tends to deteriorate unevenly over time and this indices can demonstrated this fluctuation with low individual difference [5].

In our present study, we developed a multistage CHF risk assessment model. The work presented in this paper involves the following contributions:

1. We creatively establish a four-level risk assessment model for CHF detection and quantification, including no risk (normal people, N), mild risk (patients with NYHA I-II, P1), moderate risk (patients with NYHA III, P2), and severe risk (patients with NYHA III-IV, P3).
2. We extract dynamic HRV measurements to improve the precision of the model, especially in disease quantification. These dynamic HRV measures better reflect the autonomic function change during different daily activity for individuals with CHF.
3. We apply the decision-tree-based support vector machine (DT-SVM) classifier to take advantage of SI and DI in CHF detection and quantification, respectively. We improve the performance of the classifier by integrating backward elimination (BE) with significance difference.

## Method

**Fig 1:**
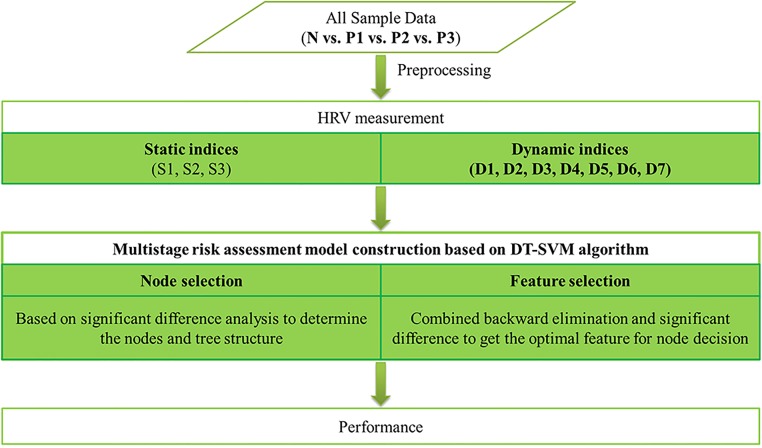
Flowchart of entire work. N: normal people; P: CHF patients, in which 1 is of NYHA I-II, 2 is of NYHA III, 3 is of NYHA III-IV; S1: basic measures of 24-h RR interval data, which reflect long-term data variation); S2: basic measures of the second 5-min segment, which representing a stable measurement condition of short-term data; S3: mid-value of basic measures of 5-min segments, which showing an intermediate state of short-term data; D1: mean value of basic measures of 5-min segments, for robustness improvement; D2: standard deviation of each basic measure of 5-min segments; D3: root mean square of each basic measure of 5-min segments; D4: coefficient variation of each basic measure of 5-min segments; D5: percentage of abnormal value (value intervening M±S) of each basic measure of 5-min segments; D6: sample entropy of each basic measure of 5-min segments; D7: fuzzy entropy of each basic measure of 5-min segments.; DT-SVM: decision tree based support vector machine.

Fig 1 presents a flowchart of the entire work.

### Data

We obtained the data used in this work from the widely-used MIT/BIH database in PhysioNet [16]. All subjects provided informed written consent. The study was approved by the Institutional Review Boards of Beth Israel Deaconess Medical Center (Boston, MA) and the Massachusetts Institute of Technology (Cambridge, MA). We chose four RR interval databases, obtaining 116 nominal 24-h RR interval records: 72 normal person samples (N, aged 20 to 76) and 44 CHF patient samples (P, aged 22 to 79). The data of normal people came from two databases: the MIT/BIH Normal Sinus Rhythm Database and the Normal Sinus Rhythm RR Interval Database [16]. The data of the CHF patients came from the Congestive Heart Failure RR Interval Database and BIDMC Congestive Heart Failure Database [17]. These records were all manually reviewed and corrected by experts.

All samples were grouped into four stages according to severity:

- 72 normal people labeled as no risk (N, aged 54.62±16.03 years);
- 12 patients in NYHA I-II labeled as mild risk (P1, aged 52.5±14.25 years);
- 17 patients in NYHA III labeled as moderate risk (P2, aged 57.24±9.28 years);
- 15 patients in NYHA III-IV labeled as severe risk (P3, aged56±11.50 years with one sample unknown).

Patients in group P3 were receiving medical therapy; therefore, we considered it as a special type with a higher risk of mortality different from groups P1 and P2. The subjects’ gender information was partially abridged, so there was no description about gender. All these data can be downloaded online from http://www.physionet.org/cgi-bin/atm/ATM [16] for free.

Before feature extraction, we preprocessed all these data:

1. deleting the first and the last RR interval;
2. excluding RR intervals longer than 3 seconds [6];
3. dividing the 24-h data into multiple 5-min segments saved in sequence.

The first two steps were performed in case of unstable measurement conditions and artificial error. The third step was for feature extraction.

### HRV Measurement

In this study, dynamic and static indices of HRV measurement were analyzed from 116 preprocessed RR interval data, both 24-h and 5-min segment RR intervals. This analysis processing included two steps: classical HRV measurement calculation and our HRV measurement calculation.

***1) Classical HRV measurement calculatio*n**: After preprocessing, we had two types of data: nominal 24-h RR interval records and 5-min segment RR interval data. With these two types of data, we calculated 18 classical HRV measurements, which included:

**Time Domain (T1~T5)**: average of RR intervals (T1); standard deviation of RR intervals (T2); root mean square of successive RR interval difference (T3); percentage of successive RR interval difference larger than 50ms (T4) [6]; coefficient variation (ratio of T2 to T1) of RR intervals (T5) [18];
**Frequency Domain (F1~F4)**: power of RR intervals in 0.04–0.15 Hz (F1); power of RR intervals in 0.15–0.4 Hz (F2); ratio of F1 to F2 (F3); total power (F4) [6];
**Nonlinear (E1~E9)**: low frequency wavelet entropy (E1); high frequency wavelet entropy (E2); normalized low frequency wavelet entropy (E3); normalized high frequency wavelet entropy (E4); ratio of E1 to E2 (E5); total power wavelet entropy (E6) [12]; approximate entropy (E7); sample entropy (E8) [19]; fuzzy entropy (E9) [20].

The time/frequency domain HRV measurements in our work followed International Guidelines [6], and frequency domain HRV measurement was calculated based on Fast Fourier Transform. The nonlinear HRV measurements are fully introduced in literature [12, 19 and 20]. The 18 Classical HRV measurement were calculated for both 24-h and 5-min segment RR intervals as ***basic measures (T1~E9)***.

***2) Our HRV measurement calculation***: Based on the classical measurements, two types of HRV measurement SI and DI were calculated, as we defined hereinafter.

The SI was calculated from HRV measurements of data in a period (5-min/24-h). This series of indices demonstrated the global or average level of cardiovascular autonomic activity, composed of four series with a total of 54 (= 18 basic measures*3 series) indices:

**S1:** basic measures of 24-h RR interval data, which reflect long-term data variation (S1T1~S1E9);
**S2:** basic measures of the second 5-min segment, which representing a stable measurement condition of short-term data (S2T1~S2E9);
**S3:** mid-value of basic measures of 5-min segments, which showing an intermediate state of short-term data (S3T1~S3E9).

In contrast, the DI was calculated from 5-min segments’ basic measures in the nominal 24-h data to evaluate the dynamic changes of symptoms and autonomic function during different activities. Here, we analyzed each basic measure of 5-min segments from 6 aspects; thus, DI has six series with a total of 126 (= 18 basic measures*7 series) indices:

**D1:** mean value of basic measures of 5-min segments, for robustness improvement (S4T1~S4E9).
**D2**: standard deviation of each basic measure of 5-min segments (D1T1~D1E9);
**D3**: root mean square of each basic measure of 5-min segments (D2T1~D2E9);
**D4**: coefficient variation of each basic measure of 5-min segments (D3T1~D3E9);
**D5**: percentage of abnormal value (value intervening M±S) of each basic measure of 5-min segments (D4T1~D4E9);
**D6**: sample entropy of each basic measure of 5-min segments (D5T1~D5E9);
**D7**: fuzzy entropy of each basic measure of 5-min segments (D6T1~D6E9).

Thus, 180 HRV measures, comprising 54 SI and 126 DI, were extracted from 72 normal person samples and 44 CHF patient samples.

### DT-SVM Algorithm based Multistage Risk Assessment Model Construction

**Fig 2:**
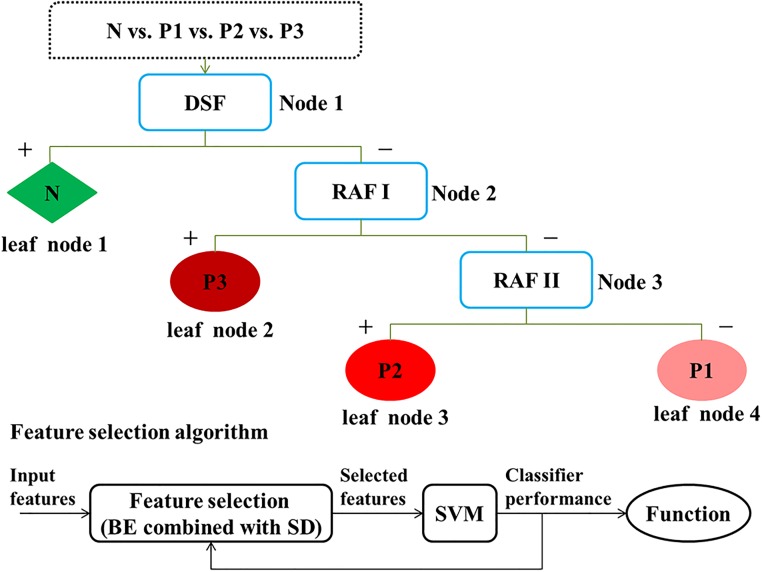
Multistage classification algorithm based on DT-SVM for risk assessment. Upper diagram: tree-structured classifier. Lower diagram: wrappers for feature selection. N: normal samples; P: CHF patients, in which 1 is of NYHA I-II, 2 is of NYHA III, 3 is of NYHA III-IV; DSF: disease screening function; RAF: risk assessment function, in which I is for discriminating the higher risk from the lower risk, II is for distinction of moderate risk and mild risk; BE: backward elimination; SD: significance difference.

In our work, we constructed a multistage risk assessment model for CHF detection and quantification (shown in Fig 2) based on the DT-SVM algorithm.

DT-SVM is an effective way of combining an SVM and a decision tree for solving multi-class problems [21]. It is a modified method of the classical SVM for dealing with its difficulty in multi-class problems, but DT-SVM brings another danger: cumulative error. This error is caused by sample misjudgment at the upper node of the decision tree and lasts throughout the rest of the classifier without elimination. The main idea of this algorithm is the conversion of multiclass classification problems into multilevel binary classification problems. Each level includes two nodes to be classified, and each node includes one or several classes. At every node, a decision is made to assign the samples. This step is repeated until all the samples reach a leaf node, to which only one class of samples is assigned. In this way, a hierarchy is formed [22].

In our research, DT-SVM was applied to establish the tree-structure of the risk assessment classifier. Performance of the classifier was determined by the tree structure and node decision [22], which depended on the nodes and input features selection.

***1) Node Selection*:** Usually, there are two kinds of tree structures: balanced or unbalanced tree architectures [22]. Furthermore, the most separable classes should be separated at the upper nodes [21]. In this study, one-way analysis of variance (one-way ANOVA) was used to calculate the significance difference of indices among two-groups [23] as separability measurement [22] on SPSS software (version 19, SPSS Inc., Chicago, IL, USA). The rule of node selection at each level is that a larger number of features in a smaller *p* value scale indicate a higher separability for binary separation. The corresponding pair of groups was used as the suitable nodes for the level.

In our work, the samples were divided into four stages, corresponding to a four-leaf-node tree. The nodes for a level were selected as follows:

First, we denote the feature set as the data set {*X*(*i*): *i* = 1,2,…,*N*} and *N* as the feature number. We then define the significance differences of *X(i)* as *P(i)*, which are calculated by one-way ANOVA between all possible two-groups, where

P(i)={p(i,k),k=1,2,…,M}, (1)

where *p(i*,*k)* represents the *p* value of the *i*th feature for the *k*th pair. In this paper, *M* is 7 for level 1 (the seven pairs are shown in Node and Feature Selection Part).

Then, we define the number of features in the particular significance value range as *count*:

$$count(k)=\sum_{i=1}^{N}d(i,k),$$ (2)

where *d* is the sign function discrimination matrix:

$$d(i,k)=\{\begin{array}{cc} 1, & \text{if }p(i,k)\leq \varepsilon ,\\ 0, & \text{if }p(i,k)>\varepsilon , \end{array}$$ (3)

and *ε* is the particular significance value range. In this paper, *ε* is initially 0.001.

Finally, we define *num* as the maximum value of *count*:

num=max(count). (4)

The pair corresponding to *num* yields the selected nodes for the level. If the maximum value associates with more than one pair, we repeat this procedure, sequentially changing the value of *ε* to 0.01, 0.05, and 0.1, until only one pair is determined for the level.

We iterated these steps for the remaining levels until each group consisted of only one class; thus, the tree structure was determined (shown in Fig 2).

***2) Feature selection*:** Owing to their high correlation, directly using all features for classification might not give the best performance [24]; thus, using an appropriate selection method for feature subset discrimination improves classifier performance. We applied BE [25] into feature selection at each node (shown in Fig 2).

First, we performed feature prescreening to remove invalid characters and improve algorithm performance. The significance level was computed among three pairs (shown in Fig 2). Considering the physiological rule, features with a high significance level (i.e., *p*>0.1) were rejected; additionally, SI were used for disease detection and DI for quantification.

Then, we applied backward feature selection to the prescreened features (shown in Fig 2). The BE algorithm begins with all features and iteratively removes them one-by-one until the remaining features reach the highest precision. The feature selection was based on the following iteration below.

We denote {*X*(*i*,*j*): *i* = 1,2,…,*M*; *j* = 1,2,…,*N*} as the feature matrix, in which *M* and *N* are the numbers of samples and features, respectively. The samples are labeled by *y*_*i*_ ∈ {-1,1}. We define *r* as the rate of training set and testing set and then select the training and testing set from *X(i*,*j*) randomly in proportion. We define the line numbers of training and testing set in the feature matrix as *tr* and *te*, respectively.

We define *Y*^*m*^ = {*X*(*i*,*k*),*i* = 1,2,…,*M*;*m* ∈{0,1,2,…,*N*}} as submatrices of the feature matrix, where *m* represents the *m*th feature deleted from the feature matrix. Thus, N+1 submatrices are formed, where *k* is the volume number of remaining features. Here, *Y*^*0*^ is the feature matrix without deletion.

Thus, the training and testing sets are defined as *Training*^*m*^ and *Testing*^*m*^:

$$Training^{m}=Y^{m}(tr,k)\;\forall tr\in \left\{1,2,\ldots ,M\right\},$$ (5a)

$$Testing^{m}=Y^{m}(te,k)\;\forall te\in \left\{1,2,\ldots ,M\right\}.$$ (5b)

We input the training set into the SVM classifier and validate with testing set. Here, we regard the SVM as a black box to score different feature combinations according to their predictive power [26]. We define the accuracy of the testing subset as *ACC*^*m*^, in which:

$$ACC^{m}=\frac{\begin{array}{ccc} number(correctly & classified & samples \end{array})}{\begin{array}{cc} number(all & samples) \end{array}}.$$ (6)

We define *S* as the difference of *ACC*^*0*^ and the maximum of the other *ACC*^*m*^:

$$S=ACC^{0}-\text{max}(ACC^{1},ACC^{2},\ldots ,ACC^{N}).$$ (7)

If *S* > 0, the algorithm ends, and the final accuracy is *ACC*^*0*^; however, if *S* ≤ 0, we need to refresh the feature matrix to the associated submatrix of max(*ACC*^1^, *ACC*^2^,…,*ACC*^*N*^) and continue the iteration.

The submatrix of the highest accuracy is the optimal feature subset. We repeat this process for the remaining levels. With the selected optimal subset, we have built the hierarchical model for CHF detection and quantification.

Finally, we have achieved the risk assessment model. The decision hyperplane functions of the three nodes were calculated. We define the hyperplane function for node decision as *f(x)*:

$$f(x)=\omega^{T}x+b,\{\begin{array}{ll} \text{if} & f(x)>0,\text{then}\;x\in class1,\\ \text{if} & f(x)<0,\text{then}\;x\in class2, \end{array}$$ (8)

Where **ω** and **b** are the weight vector and the bias that maximize the margin [27], respectively; **x** is the feature vector.

The weight vector **ω** and bias **b** are decided by the Lagrangian function:

$$J(\omega ,b,a)=\frac{1}{2}\omega^{T}\omega -\sum_{i=1}^{M}a_{i}[y_{i}(\omega x_{i}+b)-1],$$ (9)

Where *a*_*i*_ is the Lagrange multiplier and *x*_*i*_ is the training set. The weight vector and bias are determined from the partial derivative of the function *J* as

$$\omega =\sum_{i=1}^{M}a_{i}y_{i}x_{i},$$ (10)

$$b=-\frac{{\text{max}}_{i:y_{i}=-1}\omega^{T}x_{i}+{\text{min}}_{i:y_{i}=1}\omega^{T}x_{i}}{2}.$$ (11)

We named the resulting hyperplane functions according to their roles: disease screening function (DSF; for separating normal heart function from abnormal heart function), risk assessment function I (RAF I; for discriminating the higher risk from the lower risk), and risk assessment function II (RAF II; for distinction of moderate risk and mild risk). During this part, the SVM operated under a linear kernel with the same parameters.

### Validation and Performance

In our work, we randomly divided the data into training and testing sets at a ratio of approximately 1:1 (i.e., 57 samples for the training set and 59 samples for the testing set). To measure the performance of the tree-structured multistage classifier for risk assessment, we used confusion matrixes (CM) [28]. From these matrixes, we computed the widely used parameters [6] for binary classification to make a comparison with others. Calculation were performed on software MATLAB 7.11.0 (version R2010b, The MathWorks, Inc., Natick, MA, USA)

## Results

### Feature Performance Analysis with C-SVM

With the static and dynamic HRV indices introduced in Method Section, we tested their performance in 4-level risk assessment with linear kernel classical support vector machine (C-SVM) in Table 1. The input features were SI, DI and SI+DI, all with *p* value under 0.1. Feature selection method of the two classifiers was the same, i.e. the backward elimination method. The accuracy rate of C-SVM classifier was close under different input feature combination, all under 80%. Results were only 76.27% in 4-level risk assessment of CHF with SI/SI+DI inputted; while only DI inputted, the result was even 10% lower.

**Table 1:** Classification performance of classical SVM in 4-level risk assessment.

Method	Input Feature *	Accuracy (%)
Classical SVM	SI	76.27
	DI	67.80
	SI+DI	76.27

SI: static indices; DI: dynamic indices;

* represents that significance value of features were under 0.1.

While taking apart 4-level risk assessment into disease detection (N vs. P) and disease quantification (P1 vs. P2&P3), our features presented different from above. Table 2 compares our results with SI and DI in disease detection and quantification based on C-SVM classifier. Performance with different feature combinations was introduced in the table among different pairs under the linear kernel SVM. The *p* values of the input features in Table 2 were lower than 0.1, and all the features were selected with the BE method. For the discrimination of normal people and CHF patients (Table 2: N vs. P), the CHF detection accuracy of SI was 98.31%, which was higher than DI and DI + SI by over 11% and 8%, respectively.

**Table 2:** Performance of different feature combinations for disease detection and quantification.

Groups	Accuracy			Destination	Method
	SI*	DI*	SI* + DI*		
N vs. P	**98.31**	86.44	89.83	**Disease detection**	C-SVM
P1 vs. P2&P3	73.91	**91.30**	**91.30**	**Disease quantification**	

N: normal samples; P: CHF patients, in which 1 is of NYHA I-II, 2 is of NYHA III, 3 is of NYHA III-IV; SI: static indices; DI: dynamic indices;

* represents that significance level of features were under 0.1; C-SVM: classical SVM.

In contrast, prominent diversity existed when distinguishing between higher risk (P2&P3) and lower risk (P1) CHF. The disease quantification accuracy was 91.30% with DI or DI + SI inputs, but the accuracy of SI dropped by nearly 20% from the omission of DI.

### Multistage Risk Assessment Model Construction based on DT-SVM

On account of feature performance analysis result, 4-level risk assessment model was constructed under DT-SVM algorithm and significance analysis, which was described in Method Part. For each binary SVM of DT-SVM, linear kernel and other default parameter was the same. Thus, DT-SVM classifier construction included two parts: node and input feature selection and model construction, as below.

#### Node and feature selection

Table 3 shows the results of node selection based on the significance difference analysis. For the first node, there were seven possible pairs, and it was apparent that the majority of features were extremely significant between the normal person samples and the patient samples (N vs. rest, 57 features with *p*<0.001) among all these possible pairs. The number of features of this pair was larger than that of all other pairs at each significance level range (79 with *p*<0.01, 98 with *p*<0.05, and 105 with *p*<0.1). Thus, the first node decision was between normal people and CHF patients (N vs. rest).

**Table 3:** Result of node selection for level 1 among all samples.

Node	number of *p*<0.001	number of *p*<0.01	number of *p*<0.05	number of *p*<0.1	number of *p*>0.1
N vs. rest	**57**	**79**	**98**	**105**	75
P1 vs. rest	0	17	39	51	129
P2 vs. rest	15	41	73	86	94
P3 vs. rest	28	52	68	83	97
N&P1 vs. rest	48	65	88	102	78
N&P2 vs. rest	29	51	71	86	94
N&P3 vs. rest	33	61	85	97	83

N: normal samples; P: CHF patients, in which 1 is of NYHA I-II, 2 is of NYHA III, 3 is of NYHA III-IV.

Based on the result of node 1, Table 4 shows the numbers of features at different significance level ranges between the three possible pairs for node 2. The higher risk (P3) patients had one feature with *p* < 0.001 corresponding with the lower risk (P1 & P2) patients and no other pairs had this extremely significant feature. In other ranges of *p* values, the separability of P3 also performed well. Thus, the second node decision was made between higher risk and lower risk (P3 vs. rest) patients. Therefore, the third node decision was between P1 and P2.

**Table 4:** Result of node selection for level 2 among CHF patients.

Node	number of *p*<0.001	number of *p*<0.01	number of *p*<0.05	number of *p*<0.1	number of *p*>0.1
P1 vs. rest	0	3	19	39	141
P2 vs. rest	0	0	7	22	157
P3 vs. rest	**1**	**9**	**35**	**49**	131

P: CHF patients, in which 1 is of NYHA I-II, 2 is of NYHA III, 3 is of NYHA III-IV.

**Fig 3:**
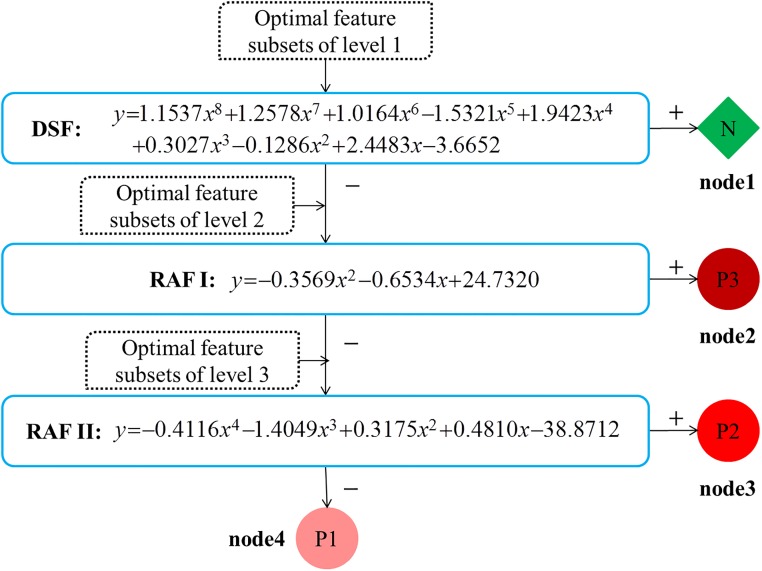
Multistage risk assessment model of CHF. DSF: disease screening function to detect normal from patients; RAF: risk assessment function, in which I is for discriminating the higher risk from the lower risk, II is for distinction of moderate risk and mild risk; N: normal samples; P: CHF patients, in which 1 is of NYHA I-II, 2 is of NYHA III, 3 is of NYHA III-IV.

Overall, an unbalanced tree was formed under our rules (shown in Fig 3) for multistage risk prediction of CHF. The first node was a binary tree between normal people and CHF patients (N vs. P); the second node was a binary tree between higher risk and lower risk (P3 vs. P2 & P1); the third was a binary tree between moderate risk and mild risk (P2 vs. P1).

The selected features for decisions of each node are shown in Table 5. Input features for each node are in sequence from SI with a *p* value under 0.1, DI with a *p* value under 0.1, and SI + DI with a *p* value under 0.1. With the aforementioned feature selection method (Method Part), the optimal features were selected from the input. The effectiveness of features also showed that a portion of the input features could fully represent them all with relatively low percentages of 7.21%, 4.08%, and 33.33%. During the feature selection of the first node, we found a subset with 23 features that provided maximum discrimination power (i.e., the accuracy was 100%) in classification between N and P. Considering the computational cost, the number of input features of node 1 (*S1T1*, *S1T5*, *S1F4*, *S2T1*, *S3T1*, *S3T3*, *S2F3*, and *S3E8*) was chosen to be similar to the other nodes (shown in Table 5). These features were fully described in Method.

**Table 5:** Selected optimal feature subsets for each level with backward elimination.

Node	Input feature numbers	Optimal feature subsets *	Effectiveness (%)
Node 1	111	S1T1,S1T5,S1F4,S2T1,S3T1,S3T3,S2F3,S3E8	7.21
Node 2	49	D5F4,D5E1	4.08
Node 3	12	D2T4,D3F3,D4T1,D5E5	33.33

*: Meaning of features were defined in HRV Measurement; Effectiveness is ratio of number of selected features to number of input features at each node.

#### DT-SVM based 4-level Risk Assessment Model

As the tree structure and optimal feature subsets of each node had already been obtained, it was easy to construct the final classification model. Fig 3 is the unbalanced multistage classification model of CHF with hyperplane functions, with input and outputs at each binary choice. These functions were described in Method Part.

All these functions were computed by SVM with the same linear kernel. The three calculated hyperplane functions are shown in Fig 3:

DSF:y=w1*X−3.6652 (12)

RAF I: y=w2*X+24.7320 (13)

RAF II:y=w3*X−38.8712 (14)

where the parameters were:

*w*1 = [1.1537,1.2578,1.0164,−1.5321,1.19423,0.3027,−0.1286,2.4483];
*w*2 = [−0.3569,−0.6534];
*w*3 = [−0.4116,−1.4049,0.3175,0.4810].

The parameter *X* is the input feature subsets, as showed in Table 5; the parameter *y* is the output value of the function, for which *y* > 0 indicates that the output type is positive (+) and vice versa.

### Validation

**Fig 4:**
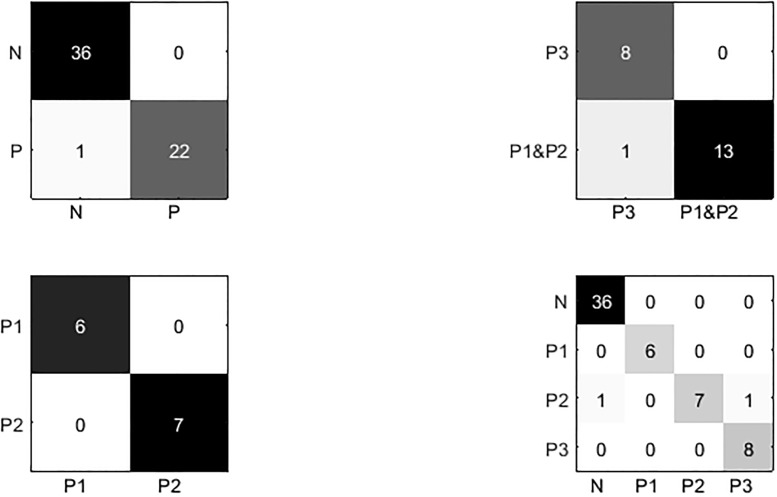
Confusion matrices. N: normal samples; P: CHF patients, in which 1 is of NYHA I-II, 2 is of NYHA III, 3 is of NYHA III-IV.

The performance of the DT-SVM-based multistage classifier for 4-level risk assessment of CHF is shown in Fig 4 and Table 6. Fig 4 shows the CM of the three binary trees and the multistage classifier. According to the CM, classification error only occurred at the classification of moderate risk (P2), in which one was misclassified as N at node 1 and one misclassified as P3 at node 2.

**Table 6:** Classification performance.

Node	TP	TN	FP	FN	ACC (%)	SEN (%)	SPE (%)	PRE (%)	AUC (%)	Total ACC (%)
Node1	36	22	0	1	98.31	97.3	100	100	98.65	**96.61**
Node2	8	13	0	1	95.45	88.89	100	100	94.45	
Node3	6	7	0	0	100	100	100	100	100	

TP: true positive, TN: true negative, FP: false positive, FN: false negative;

*ACC* = (*TP* + *TN*)/(*TP* + *TN* + *FP* + *FN*), *SEN* = *TP*/(*TP* + *FN*), *SPE* = *TN*/(*TN* + *FN*), *PRE* = *TP*/(*TP* + *FP*), *AUC* = 1/2(*SEN* + *SPE*).

Table 6 contains some common measures computed from the CM—accuracy (ACC), sensitivity (SEN), specificity (SPE), precision (PRE), and area under the curve (AUC). With the DT-SVM algorithm and backward feature selection method, we achieved a high total accuracy of 96.61%. The accuracies of nodes 1, 2, and 3 were 98.31%, 95.45%, and 100% respectively. Moreover, the precision (PRE) of each node was 100%, which demonstrated a lack of false positives. Meanwhile, the values of AUC are over 90% for every node.

## Discussion

In this paper, we applied DI and SI of HRV measurements to construct a multistage risk assessment model of CHF. This model integrated a DT-SVM classifier, the backward feature selection method, and significance difference analysis. The final calculated hyperplane functions (shown in Fig 3) achieved a total accuracy of 96.61%.

### Comparison with Others

As the changes of HRV are influenced under interact of nervous and humoral regulation, autonomic nervous system is part of the reason [29]. Mortara et al. found that static HRV was significantly lower in CHF patients with abnormal heart autonomic nerve function [30]. The adverse change of autonomic function in CHF patients was confirmed [10]. Autonomic nerve dysfunction may increase the incidence of sudden cardiac death in patients with CHF by altering ventricular electrophysiology (correlation with HRV) [31]. We speculated that CHF related to autonomic nerve abnormality. HRV measurements have been confirmed as a noninvasive tool for assessing autonomic nerve function [32]. Thus, the CHF risk assessment model of HRV proposed in this paper can serve as a noninvasive and reliable predictor of the incident risk of CHF.

Table 7 highlights the comparison with related studies. Yu et al. applied a SVM classifier and GA into CHF detection based on static (bi-spectral HRV) analysis and achieved an accuracy of 96.38% [14]. Isler et al. utilized static HRV measurements with a KNN classifier a KNN classifier for CHF detection, resulting in an accuracy of 96.39% [12]. These studies demonstrated that static HRV measurements could distinguish CHF patients from normal people with accuracies of more than 95%. However, Melillo et al. first attempted to distinguish low risk CHF patients from higher risk ones with a relative low accuracy (i.e., 85.4%) [15]. Question that arose then was what causeed the low prediction accuracy? Here we analyzed in two aspects: features (static indices vs. dynamic indices) and algorithm (C-SVM vs. DT-SVM) in our paper.

**Table 7:** Highlight.

Reference	Classes	Samples*Time	Feature	Feature Selection	Classifier	Accuracy	Highlight
Yu et al.	**N vs. P**	83*68min	SI	GA	SVM	96.38%	CHF detection based on bi-spectral HRV analysis and genetic algorithm
Isler et al.	(**N vs. P**	83*5min	SI	GA	KNN	96.39%	CHF detection by combining classical HRV with wavelet entropy measures
Melillo et al.	**P1 vs. (P2&P3)**	44*24h	SI	ESM	CART	85.40%	2-level CHF quantification in patients with CHF via long-term HRV and CART algorithm
Our work	**N vs. P1 vs. P2 vs. P3**	116*24h	**SI,DI**	BE	**DT-SVM**	**96.61%**	**4-level CHF detection and quantification using dynamic HRV measures and DT-SVM algorithm**

N: normal samples; P: CHF patients, in which 1 is of NYHA I-II, 2 is of NYHA III, 3 is of NYHA III-IV; SI: static indices; DI: dynamic indices; GA: genetic algorithm; ESM: exhaustive search method; BE: backward elimination; SVM: support vector machine; KNN: k-nearest neighbor; DT-SVM: decision tree based support vector machine.

### HRV Measurement Analysis

According to NYHA classification [5], the severity of heart failure is related to the severity of symptoms. For example, NYHA I: no symptoms and no limitation in ordinary physical activity; NYHA III: marked limitation in activity due to symptoms, even during less-than-ordinary activity; NYHA IV: experiences symptoms even while at rest. After diagnosis, the heart conditions of certain classes of patients fluctuate according to the treatment, physical condition, etc. [6].

It has been recognized that autonomic imbalance happens in heart failure, which leads to further worsening of the condition. Thus, autonomic dysfunction in CHF patients was confirmed [1]. To this end, HRV analysis of SI has already served as a powerful tool in autonomic nerve function assessment. However, we presumed that static HRV measurements could not fully reflect the fluctuation of autonomic nerve function over time among different classes of patients. As a result, Melillo et al. only reached an accuracy of 85.4% for 2-level disease quantification when using static HRV analysis [15]. Thus, in this work, we proposed dynamic HRV measurements to rate CHF risk. Comparing performance of SI and DI in disease detection and quantification based on C-SVM classifier with previous research, we can conclude that:

1. For the discrimination of normal people and CHF patients (Table 2: N vs. P), the CHF detection accuracy of SI was higher than DI and DI + SI. Based on this result, we can conclude that DI does not help in this respect. Furthermore, static HRV measurements have been proven by former studies to be reliable for high-accuracy detection of CHF [11], [12], [13], [14], [19], [33].
2. In contrast, nearly 20% diversity existed when distinguishing between higher risk (P2&P3) and lower risk (P1) CHF from the omission of DI. This demonstrates that DI are more important in discriminating higher risk patients from lower risk ones. Furthermore, inclusion of SI does not improve performance in disease quantification.

Therefore, this analysis demonstrates that: 1) Dynamic HRV measurements have an obvious advantage over SI in disease quantification; 2) DI offer no help in disease detection; 3) Static HRV measurements have excellent performance in disease detection.

### Classifier Analysis

From the SI and DI analysis above, the multistage classifier (e.g. DT-SVM) is suitable to build the CHF risk assessment model, for using certain feature in certain purpose. We compared the performance of multistage DT-SVM classifier of our paper and classical SVM one in 4-level risk assessment in Table 1. The DT-SVM classifier revealed an excellent power in 4-level risk assessment of CHF than C-SVM classifier.

The result of C-SVM in 4-level risk assessment was not satisfied for this low precision. According to the performance of DI and SI in disease detection and quantification, a relative high precision on risk assessment was possible using combination of static and dynamic HRV measurement. This was cause by the reason that the classical SVM cannot take the advantage of SI and DI described at HRV Measurement Analysis Part into risk assessment progress, as DT-SVM did.

Review the whole work, our multistage CHF risk assessment model has the following advantages:

1. The multistage model could fully combine respective advantages from static HRV measures and dynamic HRV measures. According to the conclusion in Discussion Part about HRV measurement, SI was more suitable for disease detection and DI for disease quantification. Thus the stratified structure could make full use of this trait. In our work specific features were inputted for specific nodes: SI with p < 0.1 for disease detection and DI with p < 0.1 for disease quantification.
2. The parameter setting of multistage risk assessment model also conformed to the NYHA classification. From the perspective of physiological law, adverse change in ANS activity is a hallmark characteristic of CHF [10]. And CHF patients showed weakness in vagal mechanisms to counteract sympathetic activation [10]. This dysfunction worsens along with disease exacerbation [1]. Thus, the result that the first leaf node was N and the second was P3 was conformed to their difference in ANS function.
3. The DT-SVM classifier we applied was modified by backward feature selection algorithm combined with significance difference. Significance difference analysis helped to decide the suitable tree structure and nodes. Backward feature selection method improved the efficiency of whole classifier, with features that *p* value as under 0.1.

### Clinical Significance

The multistage CHF risk assessment model achieved an accuracy of 96.61% between predicted and actual ratings. Compared with NYHA classification according to the limitations/symptoms during physical activity [5], our multistage risk model with HRV analysis is a noninvasive and objective CHF rating method. This helps reduce diagnosis mistakes caused by various physician-related factors (e.g., limited experience, work stress, fatigue) [34], ignorance of circadian clinical features caused by unprompted modulation of autonomic activity, therapy, etc. Moreover, combining with 24-h ECG recording, our model is more universal and stable than most short-/long-term HRV measurements (e.g. [19], [33]).

The ANS can modulate the sino-aerial nodal depolarizations to adjust the needs of the body. HRV analysis with SI has been used in prior research to assess ANS function of CHF patients [10]. Relative to static HRV measurements, the dynamic HRV measurements presented in this paper could better reflect the fluctuation of autonomic nerve dysfunction during physical activity. Additionally, the modified DT-SVM algorithm can fully combine both advantages and improve performance. Autonomic nerve functions trend worse in CHF patients, and HRV dynamic analysis demonstrates this small time-scale variation. Finally, no previous studies have been tested in a comprehensive multistage model. Researchers have stated that the NYHA method remains arguably the most important prognostic marker in routine clinical use in heart failure today [35]. Thus, in view of these advantages, our multistage CHF risk assessment model could serve as a clinically meaningful outcome by providing an objective and timely assessment and rating of autonomic function change trends for CHF patients in the future, especially for those with in-home monitors.

Beyond all these benefits, our study still had some limitations. First, a larger and richer data collection is needed for further study. Moreover, dynamic HRV measurements should be analyzed and explained in future work; algorithm parameter setting needs further analysis. In the future, we plan to access an early-warning risk model with more elements (e.g., therapy, clinical state) included for survival prediction.

## Conclusion

To construct a multistage risk assessment model of CHF, we applied dynamic HRV measurements to CHF detection and quantification. DT-SVM algorithm and feature selection method based on BE and significance difference was included. The data used for this study consisted of 126 DI and 54 SI of HRV measurements obtained from 116 samples of 24-h ECG records from the MIT/BIH database. According to the study, we reached several conclusions:

1. In this paper, we build a four-level risk assessment model for CHF detection and quantification based on the DT-SVM algorithm. The model succeeded with a total accuracy of 96.61%, in risk assessment among individuals of N (no risk), P1 (mild risk), P2 (moderate risk), and P3 (severe risk).
2. We creatively proposed dynamic indices for the severity evaluation of CHF. In CHF quantification (Table 2), the DI are obviously superior to the SI, increasing the accuracy from 73.91% to 91.30%.
3. The DT-SVM–based multistage risk assessment model proposed in this work significantly improved discrimination power from 76.27% to 96.61% when compared to C-SVM. The performance of our classifier improved based on the combination of the BE algorithm and significance difference.
4. According to the analysis of SI, it was clear that SI of HRV were more powerful in disease detection than DI with an accuracy of 98.31%. This is consistent with the results of prior research regarding disease detection.

In light of these advantages, the stratifying CHF risk assessment model will be a reliable and objective prognostic marker for routine clinical application (especially daily health nursing) in the future.
